# Health

**Published:** 1880-05

**Authors:** 


### HEALTH

It is a treasure, the seasoner of all the blessings of life Without it, what can we enjoy, what can we accomplish? If we possessed all the honors of the world, all the gold which has been extracted from the mines of California, we could not enjoy them only in proportion as we have health; their value is diminished if health declines. With health, OTHER THINGS BEING EQUAL, we can accomplish almost every thing we undertake. We can travel from star to star, we can dive into the depths of the earth, explore its dark regions, and bring up the hidden mysteries which it contains.

To take such a course as will insure health to an advanced age, is a proof of wisdom. We were placed in the world to be useful; and the longer we remain in it, the more good we shall accomplish, if we are endeavoring to answer the end for which we were created. To preserve our health, or regain it if lost, is to prolong or regain life. One eminent physiologist has said that, "health is life," hence to impair the former, is to destroy the latter, and *all its pleasures*, and we lie down in a premature grave.

I have no doubt, reader, but that you are to-day, indulging the hope that you will live to the age of three score years and ten; meanwhile, you wish to enjoy life and health. Well, if life is desirable—health valuable—what consummate folly it is to trifle with them, as tho' they were worthless things! How many have let their ambition blind their judgment until their health is lost, perhaps never to be found? Many there are, who are unwilling to work only with speed; consequently, they exert every muscle and nerve, until their strength is nearly exhausted, often laying the foundation of a disease which nothing but DEATH can subdue.

"Oh but," says one, "I can't help it, I have a large family to care for, and I am obliged to work every day, and night too, sometimes." Well, work on, but remember that DEATH will summons you before long, to a final reckoning, and send you into the future world, and you must go alone; your family cannot accompany you. They must remain and struggle with an unfriendly world, without your aid or counsel. HEALTH A[illegible]ERS, investigate the laws of health then, practice them

**The forms and ceremonies of politeness may be dispensed with, in a measure, in the relations and intimacies of one's own fireside, but kind attentions, never.**

